# A case report about autism spectrum disorder and comorbidities: diagnostic and treatment difficulties in transition from adolescence to adulthood

**DOI:** 10.1192/j.eurpsy.2025.109

**Published:** 2025-08-26

**Authors:** B. F. Farkas, J. Á. Balázs

**Affiliations:** 1 Department of Psychiatry and Psychotherapy, Semmelweis University; 2 Institute of Psychology, Eötvös Loránd University, Budapest, Hungary; 3 Oslo New University College, Oslo, Norway

## Abstract

**Abstract:**

We report the case of an 18-year-old male patient diagnosed with autism spectrum disorder at age of 12. Between the age of 12 and 18 he developed anxiety and substance use disorders.

He was acutely admitted to the Department of Psychiatry and Psychotherapy at Semmelweis University due to a severe manic episode with psychotic symptoms at the age of 18. He provided a multidisciplinary treatment, including medication, support group, individual addiction consultation and rehabilitation. He became stable, currently abstinent for six months and planning to graduate.

Our case report has practical implications for physicians by raising their awareness of comorbid mental disorders in the autism spectrum and also the difficulties in the transition between adolescence to adulthood.

**Keywords:**

autism spectrum, comorbidity, transient psychiatry

**Disclosure of Interest:**

None Declared

